# Supplementary material for: Are carbon emissions trading and green financial instruments synergistic? -Comprehensive quantitative research based on content analysis
Source: PLoS One. 2024 Mar 7;19(3):e0298601. doi: 10.1371/journal.pone.0298601 (PMC10919601; doi:10.1371/journal.pone.0298601)
Supplement: S1 Data — (ZIP) [file pone.0298601.s001.zip › Data/地级市Z_F工作报告.zip!/║┌┴·╜¡/╦τ╗»╩╨/└┤╫╘╣1⁄2╓┌║┼í╛┬φ┐╦╩2╛▌═°í┐_2014.docx]

各位代表：

我代表市人民政府，向大会报告工作，请予审议，并请市政协委员提出意见。

一、2013年工作回顾

2013年，我们在省委、省政府和市委的正确领导下，深入实施“五型经济”、“五个一流”、“八项重点改革”、“十项升级创新”战略思路，紧紧依靠人大、政协的监督支持和全市人民的智慧力量，全面完成了市三届人大二次会议确定的各项任务。

——主要经济指标快速增长。国内生产总值实现1210亿元，同比增长12%；规模以上工业增加值239.7亿元，增长28.2%，用电量24.3亿千瓦时，增长18.6%；固定资产投资753.8亿元，增长31.6%；公共财政预算收入64.4亿元，增长16.2%；社会消费品零售总额407.6亿元，增长15.4%；外贸进出口总额2.6亿美元，增长59.8%；实际利用外资1.93亿美元，增长27.8%；城镇居民人均可支配收入17684元，农民人均纯收入9089元，分别增长15.6%和15.1%；乡镇财政总收入35.4亿元，增长28.4%，村级集体收入4.75亿元，增长8%。规模以上工业增加值和用电量、社会消费品零售总额、外贸进出口总额、实际利用外资增幅全省第一。

——产业项目和产业园区建设再创新高。招商引资成效显著。新开工产业项目1059个，完成投资609.4亿元，增长46.4%。其中，异地项目523个，乡镇村项目710个，分别增长59.5%和76.6%。全市11个产业园区建成面积增加18.87平方公里，新入区项目408个；建成乡镇村产业集聚区59个，新增面积10.95平方公里，新入区项目137个。

——工业经济发展质效大幅提高。新增规模以上工业企业166户，拉动规模以上工业增加值增长12%。十大产业总量持续扩张，骨干支撑作用明显，其中绿色食品、石化、医药、轻工纺织规模以上企业产值、增加值、就业人数分别占总量的78.5%、80.5%和68.6%。高新技术产业增加值增长15%，实施技改项目164个，引进和培育知名品牌34个。非公经济增加值增长14%。环保工作成效显著，节能减排超额完成省定目标。

——现代农业有力推进。战胜春涝、低温、洪水等自然灾害，粮食生产“十连增”，总产达263亿斤，获产粮大县奖励资金7亿多元。千公里科技示范带标准化产业园区540个，198个晋升为国家级高产示范片。200亩以上土地流转规模经营面积1744万亩，新组建农民专业合作社4395个，同比均翻一番，14个农机合作社被授予全国示范社。在全省第一家建立了市县乡三级土地流转网络平台。推广先进实用技术20项，新增智能水稻催芽基地21处，覆盖水稻面积65%。新引进大型畜牧产业化龙头企业15户，建设畜牧科技示范园区56个、规模养殖场442个，畜牧业增加值增长11%，畜禽饲养量及产品产量居全省前列。农业综合开发、水土流失治理成效明显，人工造林面积21.4万亩，增长3.5%。抗洪抢险取得决定性胜利！

——城乡建设质量效益明显提升。全市城乡市政基础设施建设、“三供三治”、既有居住建筑节能改造以及绿化、美化、亮化总投入45.9亿元，增长35%，公园、广场、文化体育场馆等投入4.5亿元，同比翻一番，城市品位、建设质量显著提高。城乡建设总面积1561万平方米，完成投资242.5亿元。其中，城市房地产开发改造面积695.3万平方米，完成投资110亿元。乡村建设总面积865.7万平方米，其中新型城镇化和新农村建设面积201万平方米，可入住农户2.3万户，同步完成69个重点村建设。改造农村泥草房664.7万平方米。新增集中供热面积626万平方米。新建改造国省道和农村公路528公里。实际城镇化率提高2个百分点。

——商贸经济强势突破。新续建大型市场和物流项目22个，新上外资项目6个，新增有进出口实绩企业8户，为10户企业办理了进出口备案登记。肇东大庄园、绥化士达麻业等进出口能力激增，汇源果汁、绿源亚麻等形成新的进出口生长点。新增金融机构7家，金融机构存贷款余额分别增长11.6%和23.7%。都市圈经济承接区建设全面铺开，“万村千乡市场工程”、“新网工程”覆盖城乡，“寒地黑土·绿色绥化”农产品辐射全国。

——民生和社会事业高水平建设。城镇新增就业6.78万人，登记失业率低于计划控制目标0.67个百分点。城镇职工居民各项保险、新农合、新农保参保人数持续增长。新农合人均筹资标准提高20.7%。投入社会救灾救助资金19.54亿元，85.2万城乡贫困居民和110.5万灾民受益。城市低保标准每人月提高75元，农村低保标准每人年提高463元。新增养老床位1300张，农村五保供养标准达到全省一流。对冬季室外作业的环卫工人和一线交警每天增加补助10元。保障性安居工程新开工28521套，竣工17532套，改造农村泥草（危）房70674户，保障性安居工程建设全市及市本级综合评价和泥草房改造均获全省第一，创优质工程填补历史空白，建筑安全生产零伤亡获全省地市先进。以教育、卫生绩效工资改革为代表的事业单位改革成效更加凸显。全市改善办学条件总投入14亿元，改造225所学校小火炉，新改扩建公办幼儿园39所、中小学校42万平方米，建设义务教育、特殊教育标准化学校57所，招聘教师503名；卫生投入5.92亿元，新续建医疗卫生业务用房18.8万平方米，购置大中型医疗设备310台套，引进高端和适用人才381名。投入1.85亿元，解决了27.3万农村人口饮水安全。城乡妇女创业就业和扶贫解困工作扎实开展。城乡文化市场繁荣活跃，全民健身工程和人口惠家工程深入实施。社会治安综合治理水平明显提高，预防和打击犯罪能力显著增强，安全生产、食品药品安全隐患排查治理和交通、消防等专项整治力度大。保供稳价、价格监管成效显著。

——市本级整体实力明显增强。2013年确定的50项重点民生实事有45件全部完成，其他5件因规划选址和政策调整等原因正在推进。科研路投入使用，新建三水厂和一、二水厂改造水质达到国家安全标准，新建大润发超市、果菜批发市场改制等一批大型商贸企业投入运营。房地产开发面积59万平方米，建设标准住宅小区7个。投资1.7亿元，新改建道路13.4公里，铺设排水管线15.7公里，安装路灯1102基，既有居住建筑节能改造20万平方米，改续扩建公园广场10个，养老服务护理中心投入使用。房地产市场秩序明显好转，棚改遗留问题得到妥善处理，61个棚改项目完成审计，补交税费1.25亿元，解决拖欠农民工工资8165万元，保证了5000多户回迁居民的合法权益。直属高中高考二表以上普通本科进段率超全市平均值8.2个百分点。机关和事业单位职工月人均增资383元。当年归集住房公积金3.7亿元，发放个人住房贷款2.2亿元，同比分别增长45.2%和33.2%，均高于全市平均水平10%以上。绥化经济技术开发区新续建亿元以上产业项目45个，完成投资25亿元，税费收入3.24亿元，增长30%。市本级全口径财政收入28.6亿元，增长18%，公共财政预算收入8.6亿元，增长22.6%。

政府自身建设成效显著，下放市级行政审批项目25项，“三公”经费零增长，按规定完成办公用房和公务用车清理。全市公共节能、民族宗教、外事侨务、统计、旅游、残疾人、防震减灾、史志档案、气象人防、老龄双拥等工作创新发展，取得重大进步。

各位代表！一年来的奋斗实践证明，思路决定出路、战略决定发展、力度决定速度、发展决定尊严。我们的成绩来之不易。在此，我代表市人民政府，向全市人民，向市人大代表、政协委员，向离退休老领导、老同志，向各民主党派、工商联、人民团体和社会各界人士，向驻绥部队指战员、武警官兵和中省市直各部门，向所有参与、支持和关心绥化发展的朋友们，致以最崇高的敬意和最衷心的感谢！

我们清醒地看到，全市在发展中还存在需要高度重视的问题和不足，主要是：经济总量不足，三次产业比例有待优化，税收占GDP的比重较低，财政增收基础不牢。工业主导产业链条短、支撑能力弱、科技附加值低，集约集群水平亟待提高；农业抗风险能力弱，产业化程度不高，新型经营主体质量不高，合作经营水平需进一步提升；城市基础设施投入不足，配套改革滞后，城乡建设的规划设计水平、绿化的层次品位、建设的质量标准，都有待改进；城乡贫困面仍然很大，提高城乡居民收入任务艰巨，民生改善和社会事业建设任重道远；政府自身建设尚需加强。对这些问题，我们要实事求是，深入分析，找准对策，认真解决。

二、2014年工作安排

2014年，我们要深入贯彻落实十八大和十八届二中、三中全会、中央经济工作会议和省委十一届四次全会、省委经济工作会议、市委三届五次全会精神，紧紧围绕省委“全党抓发展、重点抓经济、突出抓产业、关键抓项目”的战略部署，继续强力实施“五型经济”、“五个一流”、“八项重点改革”、“十项升级创新”战略思路，科学总结改革发展实践，更高层次地谋划推进深化改革升级发展，更高水平地提升和改善民生，为实现中心崛起、富民强市，提前建成更高水平小康社会目标奠定坚实基础。

综合考虑各方面情况，今年主要预期目标是：地区生产总值增长12%以上；固定资产投资增长30%以上；规模以上工业增加值增长25%以上；公共财政预算收入增长18%以上；社会消费品零售总额增长15%以上；外贸进出口总额增长15%以上；城镇人均可支配收入和农民人均纯收入分别增长15%以上；万元GDP综合能耗下降3.2%，氨氮和二氧化硫排放分别下降2%和3.17%。

（一）加快农村工业化新型工业化和产业园区建设

突出项目兴产业、强工增实力，延长产业链条，育壮主导产业，做强产业园区，调结构、转方式，提质量、增后劲，全面提升工业经济总量和效益。

升级发展项目产业。落实好市委提出的项目产业“十个升级”，迅速掀起新一轮项目产业建设升级年活动热潮。在数量上扩张。坚持选好项目、上好项目、建好项目，改造提升“老字号”，深度开发“原字号”，培育壮大“新字号”，鼓励支持“民字号”。省际间项目到位资金增长20%以上。全年新增规模以上工业企业80户。在质量上提升。坚持巩固、完善、发展、提高，研发、技改、整合多措并举，做活存量，增产量、扩产能、提效益。支持非公经济发展，推动特色优势产业向主导产业发展。支持骨干企业扩产增效，支持中小微企业加快成长，支持困难企业扭转困局。开工亿元以上项目占新上项目50%以上。在产业链上延长。立足优势定产业，围绕产业上项目。加强技术改造，开发新产品，购买新技术、转化新成果，弥补产业链缺失和产能不足。重点放大我市的绿色食品和石化产业优势，明确核心企业，通过专业分工、服务外包、订单生产等方式，为上下游企业创造机会，做全产业链。在产业集群上发力。坚持集约集群、优势互补，符合政策、环保安全，节能降耗、土地节约、贡献发展，经过几年的持续努力，每个县都要构建起2至3个具有县域特色、区域竞争力强的主导产业，配齐配强金融、保险、物流、研发、人力资源等要素需求，形成一批具有核心竞争力的产业集群。

突出抓好招商引资。全党抓发展、全区域上项目。要突出招商重点，坚持税收、就业、龙头、产业导向，依托资源优势，大力引进产品市场潜力好、科技含量大、附加值高、低碳清洁、资源环保安全型项目，实现有质量、有产出、有贡献、有规模、有效益的目标，推动新兴产业加快成长。要创新招商方式，专业招商、专家招商、企业招商、领导招商、造势招商、借力招商组合运用，提高项目合同履约率和诚信度。注重围绕现有经济存量数量扩张式、技术升级式、合资合作式发展上项目，充分发挥现已落地建成项目对生成新的上下游产业项目星火燎原的重要作用。要拓展招商范围，到三大城市群、项目生成地、产业转移地、科研机构、大专院校招商。理顺招商程序，按照确定线索、明确意向、最后签约的程序，抓好三次集中签约新闻发布和四次集中开工现场推进。通过主动优质服务，打造优良环境，吸引投资，确保招商成效。

提升园区承载能力。加快区域化、园区化步伐，放大异地经济效应，落实主体功能区战略，做强做大优势承载区，完善异地项目利益分成机制，重点加强县与县异地项目合作，推动市场更有效配置资源。注重加强县级、重点城镇产业园区基础设施建设，满足项目落地基本需求。围绕项目审批、开工、运行、融资、用工等环节，构筑完善的商务服务体系。

强化工业经济运行管理。在品牌打造上，农产品集中向绿色标识、寒地黑土品牌上统一，靠品牌拓展市场，强化产销对接。全市新引进培育驰名、著名、知名商标34个，培育中国和省名牌32个，培育原产地保护产品2个。在市场营销上，加大政府促销力度，招商、生产、销售一体推进，在同质同价前提下，政府优先采购地工产品。在提高企业素质上，支持企业对操作人员、管理人员、特岗人员和出资人员全面培训，增强企业家社会责任感。在提高管理水平上，落实质量兴市战略，鼓励引导企业抓好内部管理，成本逆控、企业监管、安全生产、环保控制同步推进，向管理要质量、要利润、要发展。今年，园区企业档案规范化管理全面铺开，新上项目立项、安全、消防、土地、环保等准入手续办理100%，坚决避免“先上车，后买票”。在企业融资上，搭建工业企业向金融单位通报情况平台，完善工业企业助保金平台，规范企业统计报表、财会核算，培训企业上市，支持金融产品创新，提高直接融资比重。在污染防治上，坚守绿水青山是绥化的绿色GDP，优良的生态是绥化发展的重要保障。上项目、办企业必须严守生态红线、国土红线，不能破坏生态环境，不能上产能过剩项目，不能突破政策法规底线。

（二）加速建设现代农业

突出抓农转方式，积极推进现代农业综合配套改革，大力实施绿色食品精品发展战略，提高劳动生产率和科技水平，增加农民收入，推动现代农业建设取得新突破。

加强科技示范带建设。抓住现代农业综合配套改革的机遇，争取资金、政策和项目，不断提高农业综合生产能力。充分发挥科技示范带在改革中的示范引领作用，努力实现由点到线、由线到面、由表及里、表里如一的根本变化，起到引领农民、培训干部、育壮龙头、吸引投资、聚集政策的良好作用。加大智能催芽、工厂育秧、浅翻深松、大垄双行等先进种植技术的推广应用，实施水稻“双增一百”、玉米“双增二百”科技行动，加快种业研发，放大园区辐射效应，发展科技农业、标准农业、设施农业、效益农业、产业农业、节水农业、生态农业、观光农业。科技示范园区总数达到600个，建设国家级高产创建示范片200个。

切实强化装备和基础设施建设。争取新建50个大型现代农机合作社，总数达到269个，抓好规范社、示范社建设。新建25个水稻智能催芽基地，总数达到65个，覆盖率85%。完成旱田高效节水灌溉工程53.7万亩，完成8座小型水库除险加固，完成6条中小河流综合治理，修复水库、灌涝区、堤防水毁工程76处，清理民堤300公里。

突出抓好土地有序流转合作经营。支持发展农民合作社、家庭农场、农业企业等新型农业经营主体，建立健全合作经营长效机制。尊重农民意愿，自主选择载体模式，加强示范引带和内部管理，引导各种经营主体升级发展，促进规模种植经营。加强合作社金融扶持，每个农机合作社力争流转经营土地3万亩。全市200亩以上规模经营面积达到1800万亩。

加快发展畜牧经济。以两牛一猪一羊为重点，多业并举，鼓励扶持和牛、*等附加值高的特色养殖。积极开展品种改良，推广人工授精、草原改良、青贮饲料生产，强化动物疫病防控、畜产品质量安全监管。推进规模化、集约化、现代化养殖基地、标准园区、龙头企业、交易市场建设。全市畜禽饲养总量达到2.3亿头（只），新改扩建畜牧科技示范园区70个、规模养殖场400个，实现畜牧业增加值185亿元、牧业人均收入3650元。

进一步加速农业产业化进程。积极推进粮食大市向绿色食品大市转变，狠抓绿色食品基地、加工和销售建设，发挥绿色、有机、无公害农牧产品优势，打好寒地黑土品牌。加大绿色食品认证力度，建好绥化农产品质量监测与研究中心，每个县都要有绿色食品认证基地。全市绿色食品认证基地面积达到1620万亩，其中，玉米、水稻和蔬菜绿色认证面积分别达到900万亩、410万亩和75万亩。大力发展龙头企业，推动原料主产区向加工聚集区转变。同步完善利益联结分配机制，鼓励引导龙头企业向基地延伸，直接参与养殖和加工原料基地建设。全市农业产业化龙头企业发展到155家，农副产品精深加工率达到40%左右。突出抓好农产品市场体系建设，支持组建各类农副产品协会，在市本级建设占地16万平方米的绿色食品专业物流园区，其中建设2.8万平方米的绿色食品展示交易大厅，为农产品产得出、销得畅、卖上价创造条件。

着力促进农民多元增收。农业要发展，必须发展非农产业；农民要富裕，必须减少农民；农村要繁荣，必须加快城镇化。农民增收是解决“三农”问题的核心和关键。积极推进劳动力转移，抓住国家加大扶贫开发机遇，支持农民从优化种养结构、开拓销售渠道、精深加工、终端市场等各个环节中增加收入。大力发展蔬菜产业，积极搭建蔬菜产业产销对接平台，择机举办全国性蔬菜产销对接会，北菜南运，错期生产，错季销售。每个县（市、区）都要建设自己的蔬菜园区和种植带，同步发展林下经济和食用菌生产，蔬菜和经济作物种植面积上升到总播种面积的10%以上。

全面加强生态建设。重点推进生态市建设，抓好土壤、水、空气的污染防治，强化农业面源污染防治。加强黑土地资源保护，集约节约用地，抓好水土流失综合治理。加强防护林建设，严格控制采伐，完成人工造林17万亩。

（三）推进新型城镇化和以新型城镇化为重点的社会主义新农村建设

突出建城提质量，坚持“以人为本、优化布局、生态文明、传承文化”的基本原则，注重在城镇化建设中把握经济社会发展自然演进过程和政府有序推进相结合，把握人口空间集聚、产业布局的有机结合，规划城市、建设城市、管理城市、经营城市、发展城市统筹推进。

编制好城镇化规划。以城市型城市群规划为重点，抓好城镇村布局规划、县城建设规划、新农村规划、产城一体规划等4个方面的规划，完成5个未编完的县级总体规划、8个专业规划和建设用地10%控详规划。强化设计标准，注重建筑布局、建筑风格，严把平面布局、立面、造型、色彩、体量五关，打造精品。

统筹抓好城镇化和新农村建设。城镇化是以人为核心的城镇化，要坚持城市发展让人们生活更美好，把县城作为城镇化重点，有序推进重点集镇建设，坚持撤屯、减村、并乡、建城的发展思路，合理改造旧城区、开发新城区、建设产业园区协调发展，加快城乡统筹。坚持基础建设、公益建设、商业地产、居民住房、公共服务同步推进。准确把握城镇化的规律、核心和关键，推动交通网络、公共服务、基础设施、商贸物流网络、金融网点服务向新农村延伸，规划、管理、工商、税务等部门及时跟进，促进城镇化、农业现代化和新农村建设同步发展。要算好农民进城后的支出账、政府支出账、土地账、发展账、市场和政府关系账，合理配置资源，确保投资、经营、风险、利益四个主体统一，维护政府、企业和农户三者利益公平，逐步走出一条城乡建设市场化、资金筹措多元化、资源利用商品化的融资投入新路子。要加快推进先行先试的乡镇村试点，有序启动更多的试点，确保建得成、搬得进、住得起、富得了。全市更新改造老旧供水管网117.4公里，开复工“三供三治”项目43个。修建城乡公路150公里。房地产开发改造总面积582万平方米，改造农村泥草（危）房50247户。投资1.65亿元，解决农村25万人饮水安全问题。

有序推进农村转移人口市民化。以人为核心，把促进有能力在城镇稳定就业和生活的常住人口有序实现市民化作为首要任务，解决好已经转移到城镇就业的农业转移人口落户问题。把进城落户农民纳入城镇住房保障体系，重点解决好进城农民的养老保险和医疗保险。全市实际城镇化率提高3个百分点左右。

强化城乡环境综合治理。把生态文明理念融入城镇化建设全过程。深入开展城市综合整治工作，加快构建现代化、数字化、精细化城管新模式，突出治理城市乱点乱象，坚持以绿增色、以绿添景，全市新增城市绿地面积150万平方米。坚持县、乡、村、屯四位一体，实施农村环境集中连片综合整治项目，受益人口115.8万人。

（四）创新发展乡镇村经济

突出基层壮基础，深化放大“十统十分”统分结合的双层经营体制机制，加快乡镇村基层发展建设，富民强乡。

增加乡（镇）村经济总量。培育乡（镇）村自己的经济生长点、支撑点。抓招商引资，靠产业项目生成利税、生成就业增加乡镇村经济总量，全市乡镇村项目增长10%以上；抓现代农业，靠发展各类农民合作社、提升种养业增加乡镇村经济总量；抓农民土地流转，靠农民转移就业、扩大农民财富增加乡镇村经济总量；抓“三资”管理，靠提高“三资”效益增加乡镇村经济总量。乡镇财政收入增长25%。

提高乡（镇）村管理水平。坚持“钱随事走、集中力量、形成能力、解决问题”，解决好生钱、用钱、管钱的问题。坚持村财民理乡代管，乡财乡用县监管，集中力量办大事，重点用于公共公益事业建设、民生保障和改善生产生活环境上，用于增强企业扩大再生产能力，“以钱生钱”上。突出解决好乡村两级运行困难，克服短期行为，杜绝盲目举债。

提高乡（镇）村干部素质。加强教育培训，促进广大干部察民情、接地气，更加热爱农村、熟悉农业、善抓项目、融入农民。充分发挥好群团组织和大学生村官作用，加强基层组织建设，努力建设一支忠诚服务、高度负责的队伍，一支想干事、会干事、能干成事的队伍。

（五）大力发展现代服务业

突出活贸促发展，更高层次地谋划推进都市圈城市型城市群城镇群经济，做大做强现代服务业。

提升传统服务业。加快传统服务业发展提速、质量提高、结构提升。突出发展金融、保险、证券、交通、仓储、地产等生产性服务业和餐饮、家政、养老、健身、社区服务、时尚产业等生活性服务业。发挥绥化交通优势，建设内陆港，探索内陆地区对外开放的新通道。

发展现代商贸业。积极发展专业服务业和信息服务业，推进商贸企业信息化建设。努力开拓国际市场，壮大进出口贸易企业群体，着力建设食品、服装、亚麻纺织和粮食精深加工产业的专业型外贸进出口基地。各县市区要加快引进建设大型现代商贸综合体，加快发展对外贸易，推动对俄经贸合作。全市新增进出口实绩企业5家，实际利用外资额增长15%以上。

加快现代物流业。坚持信息化引领，发展电子商务，开展网上交易，建设现代化营销网络。吸引有实力、有市场、有业绩、有信誉的高水平大型物流企业和项目落地，建立起内部物流网络。重点推进农产品配送体系和冷链物流建设，建设好各种类型的批发市场。

推进特色旅游业。深度规划打造肇东、兰西沿松花江哈尔滨近郊生态自然文化旅游带，肇东、安达、明水、望奎等市县西部草原湿地旅游带，兰西、望奎、北林等呼兰河休闲文化旅游带，海伦、绥棱、庆安小兴安岭山区旅游带，主要公路沿线现代农业旅游带，小城镇和新农村观光旅游带，农家乐、红色文化、民俗文化旅游带等。加强住宿、餐饮和游乐等设施建设管理，开发旅游产品，积极培育新的经济增长点。

（六）抓好民生和社会事业

突出科教惠民生，创建全省一流的民生和社会事业，集聚全社会力量，加大投入，让深化改革、升级发展的红利惠及城乡百姓。

让社会保障能力更强。全市新增就业岗位5.2万个，城镇登记失业率控制4.3%以内，就业困难群体就业2万人。全市职工基本养老保险、医疗保险、新农保覆盖范围继续扩大。新上企业职工实现全员参保，开展失地农民养老和医疗保险，全面完成城乡居民大病保险省级试点工作。加强城乡低保管理，应保尽保，稳定提高低保标准。实施敬老、爱老、助老工程，新建公办养老机构3家、床位500张。全市建设廉租房、公租房5612套，新增集中供热面积300万平方米。深入开展“百乡千村价格惠民行动”，建立水电油气等阶梯价格制度，实施价格补贴联动机制，提高弱势群体保障能力。

让社会事业水平更高。突出解决好农村教育、医疗发展问题，合理布局，科学定位，充实力量，激发活力。适应教育卫生发展需要，适度加强硬件建设，重点加强软件建设，推进市县乡村资源共享、均衡发展。在教育方面，巩固扩大学前教育三年行动计划成果，建设义务教育标准化学校22所。大力开展教师培训提高，有计划地招聘新教师，推进教师队伍结构优化。严格落实《中小学教师违反职业道德行为处理办法》，从严规范学校办学和教师从教行为。力争用3年左右时间完成农村教育布局调整和校车普及。在卫生方面，深化医药卫生体制改革，抓好县级综合医院执业医师培训，加快推进医院后勤社会化步伐。整顿规范医疗秩序，严厉打击非法行医。深入实施妇女儿童发展规划，人口自然增长率控制在3‰以下。加强科学技术普及，提升全民科学素养。加强基层公共文化设施建设，加快绥化广播电视台公共频道在市县区落地和新闻综合频道数字化升级。建设四级全民健身工程300处，提高体育公共服务水平。

让社会治理方式更新。严明安全生产党政同责、一岗双责、齐抓共管责任机制，做到“全覆盖、零容忍、严执法、重实效”。加强食品药品安全监管，使老百姓吃得饱、吃得好、吃得安心。坚持依法治市，加强社会治安综合治理，抓好企业和校园周边环境整治，严查严打各类违法犯罪活动，命案必破，黑恶必除，刑事案件破案率稳中有升。畅通群众利益诉求渠道，推进信访信息化、法制化建设，加大人民调解工作力度，有效维护社会稳定。加快示范社区建设，推进社区管理体制机制改革，把城乡社区建设成为社会管理的平台、居民日常生活的依托、社会和谐稳定的基础。

（七）建设法治政府和服务型政府

扎实开展群众路线教育实践活动，创新行政管理体制机制，增强政治、政策敏感性，增强政府的公信力和执行力。

深化改革，转变职能。以实施全省五大规划为切入点，深入落实市委确定的各项重点改革，牵动重要领域和关键环节改革。处理好政府与市场的关系，充分发挥市场在资源配置中的决定性作用，创新财政管理体制机制，深化政府采购和招投标改革，积极推进服务市场化。大力清理规范中介机构，让行政业务彻底退出中介。大力推进事业单位绩效改革，厘清事业单位与行政、市场的关系。更好地发挥政府作用，进一步简政放权，中央和省要求取消和下放的行政审批项目，坚决取消和下放到位;没有法律法规依据和变相保留的行政审批项目，坚决取消;依据我市情况应该和可以取消的行政审批项目，坚决取消。按照市场对资源配置起决定性作用的要求，把不应属于政府的权力还给市场和社会。

真抓实干，改进作风。认真贯彻中央和省市委关于改进作风的各项规定，坚决反对“四风”，坚持深入基层、深入实践，踏石留印、抓铁有痕。

依法行政，强化监督。紧紧依靠市委领导，主动接受人大的法律监督、政协的民主监督、媒体的舆论监督、群众的社会监督。完善听证、论证和新闻发布制度，提高科学民主决策水平。认真办理人大代表、政协委员议案、提案、意见、批评和建议，回应好人民关切。

廉洁从政，树好形象。严格执行党政领导干部廉洁从政各项规定，扎实推进惩治和预防腐败工作。敬畏并严格执行党的政治纪律、组织纪律、财经纪律、工作纪律和生活纪律，及时解决人民群众反映强烈的突出问题。

全面经营，全程管理。经营好招商引资项目，经营好现代化农业，经营好城市，经营好国有和集体资产，经营好生态，经营好环境，经营好国家和省的宏观政策。通过全面经营，提高财政收入占GDP的比重、税收占财政收入的比重、产业投资占固定资产投资的比重、非公经济产业投资占全市产业投资的比重、民生投入占财政支出的比重。守住国家宏观政策底线，坚持财税纪律底线，搞建设不能盲目铺摊子，要量力而行，尽力而为，集中财力办大事。加强预算单位资产管理，全面清理出租出借资产。严格管理好国家和省专项资金，管理好机构编制和人员，执行好财政预算，全面提高政府管理能力和管理水平。

（八）办好市本级50件民生实事

坚持率先发展，着力提升市本级实力，辐射带动全市发展。加大民生投入，全力办好50件重要民生实事和重大民生工程。

(1)落实老年人、残疾人免费乘公交政策，2014年1月1日全面实行。(2)为机关和企事业单位离退休人员及城乡低保等低收入群体发放一次性御寒补贴。(3)将环卫工人冬季清冰雪补贴从每人每天5元提高到每人每天15元。(4)将市直属学校教学黑板全部更换成电子白板。(5)加快移址新建第七中学，完成年度建设任务。(6)长江小学投入使用，新建黄河小学。(7)市妇幼保健院新址建设竣工投入使用。(8)在兴隆大家庭项目区建设1处6000平方米综合体育馆。(9)改造完善城区5个公园，把人民公园和森林公园建成集健身、休闲、娱乐、观光于一体的综合性乐园；改造建设朝中广场，增添植物园功能；改造八中旧址，建成开放式街心公园。(10)在西湖公园招商引资建设一处占地5万平方米的儿童乐园。(11)加快旧城剩余区片棚改进程，力争改造70万平方米以上。(12)加快新城区建设，放大放活收储土地效益，新增房地产开发面积60万平方米以上。(13)投资9000万元，将25公里的宝山至西长发公路改造为国家二级路。(14)投资4000万元，将4.2公里的东富至王家店收费站改造为国家一级路。(15)投资3000万元，维护改造市区道路。(16)加快城市街路照明改造，改造安装路灯1180基。(17)完成城区绿化总体规划和设计方案，梯次推进，今年安排2000万元财政绿化资金。(18)推进城南污水渠改造，建设城南河。(19)续投1000万元更新一、二水厂设备，同时加速城市二次供水改造，提高城市水质。(20)城区天然气入户新增3万户。(21)垃圾发电项目力争投入运营，彻底解决城市垃圾二次污染问题。(22)全面规划设计供热、供水、排水管网，优先改造城区西侧排水泵站，有序改造城区老旧供热管网，尽快撤完小锅炉，逐步解决困扰多年的市区排水排污问题和局部分散供热问题。(23)统一规划设计城市电网。(24)统筹规划电力、通讯光缆、电缆设施，将市区5条主要干道的地上明线逐渐改造为地下暗线。(25)在宝山镇建设农贸综合批发交易市场。(26)完善原有社区功能，新建改建12个社区用房。(27)移址新建武警营房，原址改建特警驻地和警官学校。(28)完成看守所改造建设。(29)完成数字化城市建设，打造集治安防控、交通监管、城市管理、应急处置、群众诉求等于一体的城市综合数字化平台。(30)加大市区环境治理力度，彻查清理城区私接滥建，统一规划整治市区5条主要街路牌匾，媒体公示、全民监督，逐步解决好群众关注的城市秩序混乱问题。(31)规范城区汽配修理，推进六禾新城汽配修理专区建设。(32)实施出租车、公交车管理办法，规范运营秩序，方便居民出行，打造城市新名片。(33)建立公交停车场。(34)东城客运站竣工交付使用。(35)规划设计南城客运中心，力争完成招标并施工。(36)建设烟草公司物流园。(37)在市本级新增2-3家金融机构。(38)完成多普勒气象雷达站建设。(39)辟建宝山镇到开发区沿路蔬菜科技示范带。(40)加快推进红兴水库、阁山水库、十六道岗水库建设。(41)加速推进宝山、东富城区化建设，完善提升宝山新城功能，启动东富新城建设。(42)加快推进北林区四方台镇、西长发镇等新型城镇化建设。(43)加快建设北林区东富村农民新城。(44)启动西三环路规划设计，完成立项报批。(45)启动大唐热电联产建设项目，建设城区新热源。(46)启动绥化经济技术开发区铁路专用线建设。(47)启动推进绥大高速路建设。(48)启动推进兰明青一级公路建设。(49)启动推进兰明青铁路建设。(50)启动争取海伦或绥棱机场规划论证。

各位代表，奋斗度岁月，工作结硕果！让我们在市委的坚强领导下，坚持战略、落实战略，真抓实干、锐意创新，更高层次地谋划推进深化改革升级发展，为实现中心崛起、富民强市，提前建成更高水平小康社会而努力奋斗！
